# Functional proteomics of patient derived head and neck squamous cell carcinoma cells reveal novel applications of trametinib

**DOI:** 10.1080/15384047.2022.2055420

**Published:** 2022-03-28

**Authors:** Myles Vigoda, Chase Mathieson, Nathaniel Evans, Carolyn Hale, Jennifer Jennings, Olivia Lucero, Sophia Jeng, Daniel Bottomly, Daniel Clayburgh, Peter Andersen, Ryan Li, Daniel Petrisor, Jeffrey W. Tyner, Shannon McWeeney, Molly Kulesz-Martin

**Affiliations:** a Department of Dermatology, Oregon Health & Science University, Portland, OR, USA; b Michigan State University College of Osteopathic Medicine, East Lansing, MI, USA; c Division of Bioinformatics & Computational Biology, Department of Medical Informatics and Clinical Epidemiolog, Oregon Health & Science University, Portland, OR, USA; d Knight Cancer Institute, Oregon Health & Science University, Portland, OR, USA; e Oregon Clinical and Translational Research Institute, Oregon Health & Science University, Portland, OR, USA; f Department of Otolaryngology Head and Neck Surgery, Oregon Health & Science University, Operative Care Division, Portland VA Health Care System, Portland, OR, USA; g Division of Hematology and Medical Oncology, Oregon Health & Science University, Portland, OR, USA; h Department of Cell, Developmental & Cancer Biology, Oregon Health & Science University, Portland, OR, USA

**Keywords:** Cancer, HNSCC, Trametinib, MAPK, PDX

## Abstract

In this study, we report a differential response of mitogen-activated protein kinase–kinase (MEK) inhibitor trametinib in 20 head and neck squamous cell carcinoma (HNSCC) patients’ tumor-derived cell cultures. Relatively sensitive and resistant cases to trametinib were identified using high throughput metabolic assays and validated in extended dose response studies in vitro. High throughput metabolic assays exploring combination therapies with trametinib were subjected to synergy models and maximal synergistic dose analyses. These yielded several candidates, including axtinib, GDC-0032, GSK-690693, and SGX-523. The combination regimen of trametinib and AXL/MET/VEGFR inhibitor glesatinib showed initial efficacy both in vitro and in vivo (92% reduction in tumor volume). Sensitivity was validated in vivo in a patient-derived xenograft (PDX) model in which trametinib as a single agent effected reduction in tumor volume up to 72%. Reverse Phase Protein Arrays (RPPA) demonstrated differentially expressed proteins and phosphoproteins upon trametinib treatment. Furthermore, resistant cell lines showed a compensatory mechanism via increases in MAPK and non-MAPK pathway proteins that may represent targets for future combination regimens. Intrinsic-targeted options have potential to address paucity of medical treatment options for HNSCC cancer patients, enhance response to extrinsic targeted agents, and/or reduce morbidity as neoadjuvant to surgical treatments.

## Introduction

Head and neck squamous cell carcinoma (HNSCC) is the seventh most common cancer worldwide, resulting in 450,000 deaths annually, with 10,030 deaths in the United States.^1–3^ It is estimated that. The 8-year survival of HNSCC is approximately 30.2% for HPV negative disease and approximately 70.9% for HPV positive disease.^4,5^ In this study, we focus on HPV negative disease, which is more aggressive. The standard treatment for HNSCC largely depends on tumor location, TNM staging, and medical comorbidities. Treatment incorporates surgery, chemotherapy, and radiotherapy singly and in combination. Chemoradiotherapy is the standard of care in unresectable, locally advanced disease.^4^ Thus far, two PD1 targeted immunotherapies, pembrolizumab and nivolumab, and one tumor intrinsic EGFR targeted therapy, cetuximab, are FDA approved for treatment of HNSCC.^6^

Although the introduction of new immunotherapies has greatly expanded HNSCC treatment options, only 13–18% of recurrent or metastatic cases are responsive, with all but 5% ultimately progressing to death.^4,7,8^ Additionally, cetuximab, the only tumor-intrinsic targeted therapy, was approved for use in combination with radiotherapy.^9^ However, cetuximab has declined in favorability as radiotherapy alone is no longer the standard of care.^5^ When cetuximab is used in combination with chemotherapy in the recurrent/metastatic setting, it extends progression-free survival (PFS) and overall survival for less than 3 months.^9^ Unfortunately, cetuximab also carries multiple side effects, resulting in an increase in adverse events.^5^ These limited therapeutic options represent a paucity in targeted therapeutics available for the treatment of advanced HNSCC, underscoring a need for new approaches.

MEK inhibitors targeting the MAPK pathway are used in combination with BRAF inhibitors in melanoma, metastatic lung cancer, and metastatic anaplastic thyroid cancer when a *BRAF V600E* or *V600K* mutation is present. In melanoma, trametinib single agent increased PFS by 4–7 months in *BRAF V600E* mutant tumors.^10^ Interestingly, there was a partial response rate of 10% in *BRAF* wild-type tumors, demonstrating that a subset of non-*BRAF* mutant tumors may benefit from MAPK inhibition. In HNSCC, Phase II randomized clinical trials with single-agent trametinib have shown modest results, with >25% reduction in tumor volume in 11/17 patients treated prior to tumor resection.^11^While estimates vary, the RAS-MAPK pathway has been found to be mutated in as high as 18% of HNSCC.^6^ Our underlying hypothesis is that patient-specific tumor intrinsic targeting has a role in improving therapeutic options and treatment efficacy. In this study, we focus on the more aggressive HPV negative HNSCC, examining trametinib as a therapeutic agent across HNSCC patient-derived primary and early secondary cell cultures; demonstrate patient-specific efficacy in vivo; and identify potential combination therapies that may synergize with trametinib treatment.

## Results

### Trametinib Differential Response in patient derived HNSCC Cohort

**Figure 1.**
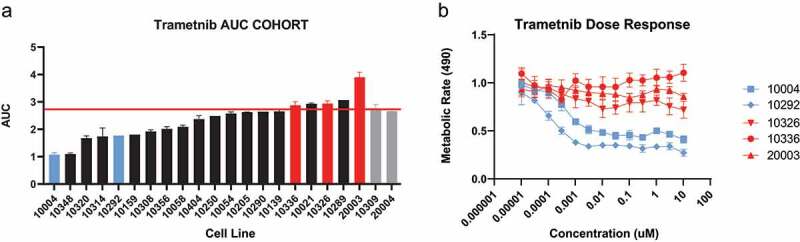
Patient derived HNSCC cells were exposed to trametinib at a max concentration of 20 μM. (a) Differential AUCs of HNSCC cohort of 20 tumors. Highlighted selected cases: Sensitive cases (blue bars), resistant cases (red bars), HNSCC cells (black bars), and tumor matched normal tissues 10309 and 20004 (gray bars). Red line demarcates no drug effect. (b) At time of selection, sensitive cases that were <20% of the median IC50 values where selected for validation. Selected cases were validated in an extended dose response assay for 2 sensitive and 3 resistant cell lines.

Primary tumor cell cultures derived from 20 HNSCC patients were subjected to a drug screen of 200+ inhibitors, natural products, and combinations (Mathieson et al., unpublished). Trametinib was identified as showing selective sensitivity and resistance in the HNSCC cohort, demonstrating a non-uniform metabolic rate profile across cases (Figure 1a). For the two cases with cell lines derived from patient-matched uninvolved mucosa, no toxicity was detected in response to trametinib (Figure 1a Supplementary Fig. S1A). Trametinib decreased the metabolic rate of patient ID 10308 tumor cells, whereas matched mucosal cell lines (10309) were largely unaffected (P value < .05) (Supplementary Fig. S1A). Among the HNSCC lines, trametinib showed a bi-modal distribution of responses across the cohort, suggesting two distinct populations of relative responders and non-responders (Supplementary Fig. S1B).

To confirm selective sensitivity, relatively sensitive cell lines 10004 and 10292 and resistant/insensitive cell lines 10336, 10326, and 20003 were further characterized using an extended dose range in triplicate experiments, confirming case-specific dose-dependent inhibition (Figure 1b). Cell line 10250 was initially characterized but was ultimately excluded from analyses due to variation in drug response at different passages. Importantly, responses reached IC50 values as low as 0.3 nM, well within a clinically achievable range.^12^ Additionally, trametinib, but not TAK-773 (a MEK1 inhibitor), showed reductions in metabolic rate, demonstrating that both MEK1 and MEK2 inhibition are necessary for cellular response at clinically relevant concentrations (Supplementary Fig. S1C). Notably, sensitive case 10292 carries an activating *HRAS* mutation. *HRAS* mutations in non-HNSCC cancer cell lines have previously shown sensitization to MAPK inhibitors.^13^ In cell line 10336, many inhibitors, including trametinib, resulted in increases in metabolic rate, highlighting the importance of stratifying patient populations by drug sensitivity.

### Trametinib drug synergy screen

After a preliminary screen of trametinib in binary combinations with 150+ other drugs and natural products, we identified and selected synergistic and antagonist drug combinations to be further validated (data not shown). The combination panel was further expanded using drugs with more specific drug interactions to characterize drug effects and pathways. To analyze the combination synergies, we used two different models for analyzing combination synergies, the Bliss independence model and the Loewe additivity model.^14^ Bliss beta coefficients were calculated using SynScreen high throughput combination software.^15^ Chou-Talalay method for analyzing combination indices yielded similar results to the Bliss beta calculations, with a − .65 Spearman correlation^16^ (Supplementary Fig. S2A, S2B). Furthermore, the Combination index (CI) methodology allowed for the identification of regimen dosing that yielded the minimum CI value across the drug dose ranges.^16^ This allowed us to identify both the synergistic drug targets and the optimal synergistic drug dosing regimens (Supplementary Table S1).

**Figure 2.**
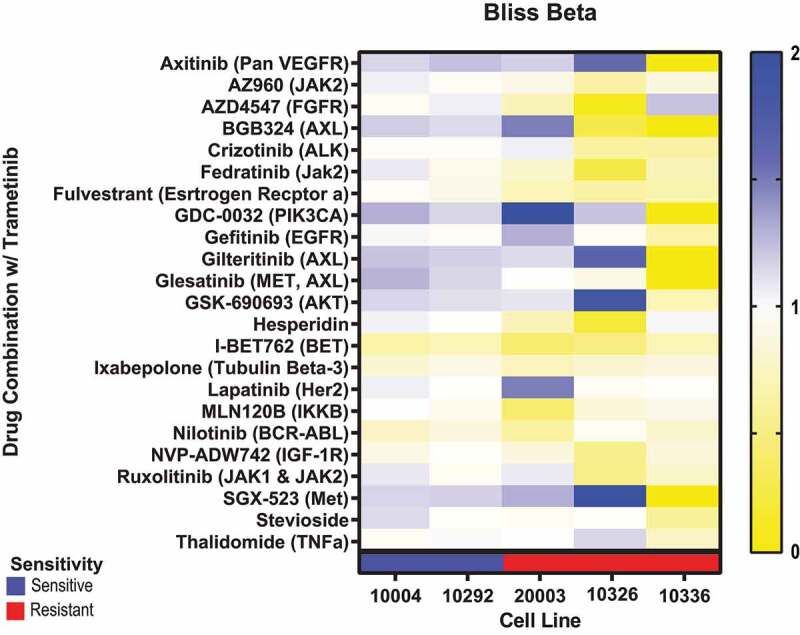
Patient-derived HNSCC tumor cells from 2 sensitive and 3 resistant lines were exposed to trametinib combination treatments in 7 × 7 drug matrices. Bliss beta values were generated for each cell line and drug combination. Bliss beta values >1 (blues) are considered to be synergistic, bliss beta values of 1 (white) are considered to be additive, and bliss beta values < 1 (yellow) are considered to be antagonistic.

The Bliss beta values show a normal distribution around the Bliss additivity value of 1, with sensitive cell lines accounting for the majority of the synergistic combinations (Supplementary Fig. S2C, S2D). Stratifying by cell line, 10336 is identified as being particularly resistant to multiple inhibitors, skewing the distribution of the resistant cell lines (Supplementary Fig. S2C). GDC-0032, a PIK3CA inhibitor, was synergistic in 5/6 cell lines, reduced metabolic rate by up to 91.5%, and sensitized 2/3 of the resistant cell lines to trametinib treatment (Figure 2 and Supplementary Fig. S2A). Also synergistic were AKTi (5/6) VEGFRi (5/6), METi (5/6) and AXLi (4/6); however, these compounds had a less pronounced effect at reducing overall metabolic rate (Figure 2).

Trametinib induced a dose-dependent decrease in the phosphorylation levels of ERK regardless of the sensitivities of the cells (Supplementary Fig. S3A). This suggests that while trametinib is effective at blocking MAPK pathway inhibition, only a subset of cells will experience a decrease in metabolism. In the sensitive line, 10004, and the resistant line, 10336, trametinib treatment induced an increase in Phosho-AKT levels (Supplementary Fig. S3B). This could partially explain trametinib synergies between PIK3CAi, AKTi, VEGFRi, METi, and AXLi, all of which either directly target or target an upstream regulator of Phospho-AKT.

### In Vivo PDX trametinib model

**Figure 3.**
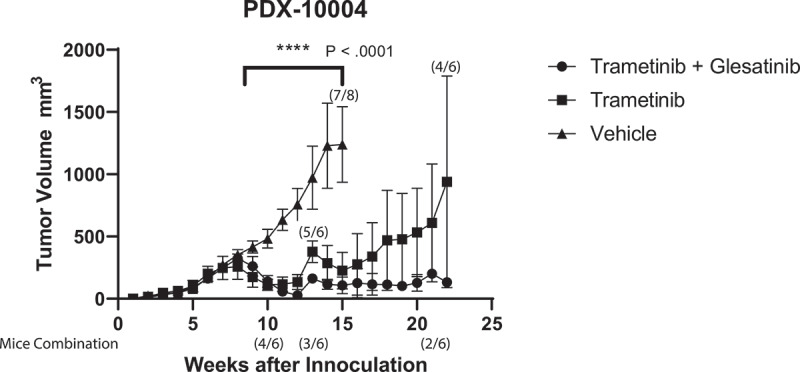
Patient-derived HNSCC tumor cells for patient 10004 were implanted and established at 350 mm^3^. Mice were treated with either trametinib (N = 6), trametinib + glesatinib (N = 6) combination treatment or vehicle (N = 8). Data represents the group mean ± SD. Change in mouse numbers displayed above data points for vehicle and trametinib groups as a fraction of the total cohort. Change in mouse number displayed below the graph for trametinib + glesatinib groups. P value shows range of statistical difference between vehicle and treatment groups.

To evaluate the relationship of trametinib sensitivity in vitro to efficacy in vivo, we established a patient-derived xenograft (PDX) model using patient-specific primary tumor cells. Trametinib significantly decreased tumor volume in 10004-PDX models (average decrease of 72% tumor volume compared to vehicle, 300 mm^3^ to 82 mm^3^) (Figure 3). While numbers of mice in the pilot study of trametinib in combination with MET inhibitor glesatinib were low, this regimen showed promising increased magnitude and durability of response (average decrease of 91% tumor volume, i.e., 320 mm^3^ to 29 mm^3^), compared to trametinib alone (Figure 3). Remission in resistance to combination treatment was durable for >20 weeks, as opposed to ~17 weeks in single-agent trametinib treatment (Figure 3). The inhibitor concentrations for the glesatinib combination (glesatinib: 60 mg/kg, trametinib: 1 mg/kg), were based on previous studies^17^ and were concordant with our in vitro analysis of dose-specific maximum synergy (Supplementary Table S1). Although glesatinib alone showed little effect as a single agent in vitro, suggesting that the combination regimen and not glesatinib as a single agent is responsible for the tumor response, the absence of a glesatinib single-agent control in vivo is limitation of our in vivo study would need to be addressed in follow-up studies (Supplementary Fig. S4).

PDX models have been shown to be reliable in terms of replication of original tumor and predicting drug sensitivities (Supplementary Fig. 5A).^18^ The relevance of the PDX model in our study is based upon several lines of evidence. The epithelial tumor cell morphology was indistinguishable in the patient-derived primary cultures and the mouse tumor derived primary culture of that patient after reestablishment of the PDX tumors in culture. Additionally, vimentin-keratin staining of the cultured cells showed the same epithelial to mesenchymal transition (EMT) characteristics in both the PDX and original tumor: 100% epithelial with 4% mesenchymal characteristics. Similarly, tumor pathology of the original case and the PDX tumor from that case were similar based upon H&E staining of tissue sections from each tumor (Supplementary Fig. S5A). Finally, the cells from the mouse and primary tumor exhibited similar drug sensitivities (Supplementary Fig. S5B).

### RPPA proteomic response in trametinib sensitive and resistant cell lines

Reverse Phase Protein Array (RPPA) was conducted in order to determine the molecularly and functionally relevant phosphoproteomic changes in response to MEK inhibition. Lysate collected from cells treated with trametinib or vehicle for 24 hours revealed that maximum ERK inhibition occurred at 100 nM regardless of the functional response to trametinib, consistent with other studies (Supplementary Fig. S3A).^19,20^ Basal proteomic levels of cell lysate showed no significant correlations with drug response.

To test the functional relevance of MEK inhibition, lysate was collected from cells treated with trametinib or vehicle at the IC50 value for 3 days. The resistant cell lines were treated at maximum dose of 312 nM. Prior to RPPA analyses, immunoblot and metabolic rate assays confirmed the expected functional and molecular responses, ensuring the integrity of the cell lines and analyses (Supplementary Fig. S6). Both 24-hour and 3-day treatment samples were collected and sent to MD Anderson for RPPA analyses.

All trametinib-treated cell lines showed a significant decrease in MAPK phosphorylation at T202 and Y204 (Supplementary Fig. S7). Although the MAPK phosphorylation antibody used for these results was designated as “use with caution” and therefore not used in the primary analysis, the decreases in MAPK phosphorylation indicates that trametinib was effective in inhibiting its primary target.

**Figure 4.**
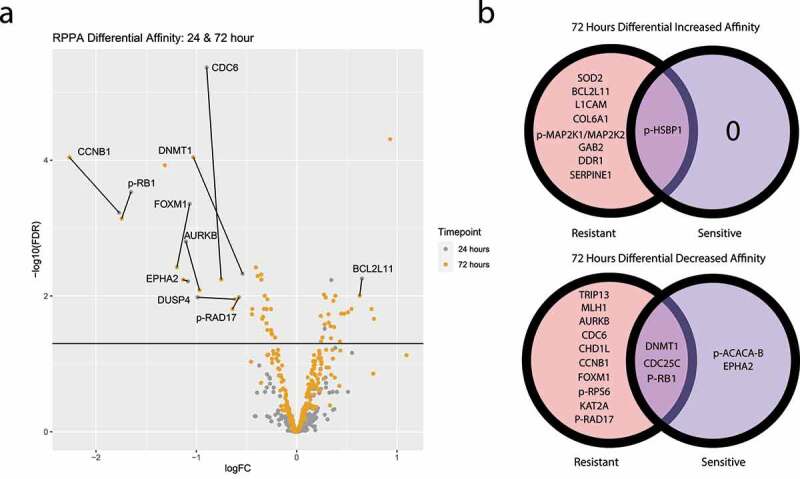
Sensitive and resistant cells were treated with trametinib for either 24 or 72 hours at 100 nM or their IC50 values, respectively. (a) Volcano plot shows log fold change of perturbed protein expression of pooled sensitive and resistant cell lines at both the 24-hour (gray) and 72-hour (yellow) time points. Proteins above the horizontal black line demonstrates statistically significant differential affinity with labeled proteins being statistically significant at both time points. (b) Venn diagrams demonstrate proteins with statistically significant increased (upper) and decreased (lower) affinity at the 72-hours after treatment with trametinib, stratified by trametinib-sensitive and trametinib-resistant.

Only MD Anderson-validated antibodies for differential affinity analyses between 24-hour and 72-hour groups (Figure 4a). The 72-hour treatment group showed significant differential affinity for 53 proteins and phosphoproteins, compared to 12 proteins and phosphoproteins in the 24-hour group, with 10 proteins overlapping between groups (Figure 4a). Interestingly, decreases in cyclin B1 and decreases in RB phosphorylation along with increases in the pro-apoptotic protein BIM suggest that trametinib is not only slowing cell cycle progression but also working to shift the apoptotic balance of the cell. There were 21 additional proteins that had increased affinity at only the 72-hour time points, including increases in targetable proteins DDR1, LCK, MMP14, PAI-1, SOD, CD26, p-AKT 1/2/3 and VEGFR2. In contrast, only NOTCH1 and targetable FAK showed increased affinity at only the 24-hour time point.

When comparing between resistant and sensitive cell lines, the resistant groups showed a greater number of protein antigens differentially expressed at both the 24- and 48-hour time points, compared to the sensitive groups (Figure 4b). Eight genes showed an increase in differential affinity at 72 hours in the resistant groups but not in the sensitive groups (Figure 4b). The resistant lines showed increased phosphorylation of MEK1 at 72 hours that was not seen in sensitive group. GAB2, which mediates receptor tyrosine kinase activation of the MAPK pathway, and DDR1 tyrosine kinase receptor, which signals through the MAPK pathway, were also differentially increased in resistant cell lines.^21,22^ Together, these proteomic changes, specific to the resistant group, are suggestive of a MAPK reactivation mechanism available to trametinib-resistant cell lines. Phosphorylated heat shock protein was differentially increased in both resistant and sensitive groups, suggesting cellular stress in both resistant and sensitive cell lines. CDC25C, DNMT1, and RB1 phosphorylation were decreased across both resistant and sensitive cell lines at 72-hours. AURKB, CDC6, CCNB1, and FOXM1 showed differential decreases in resistant cell lines at both the 24-hours and 72-hour time points but failed to reach significance in the sensitive cell lines.

## Discussion

In this study, we show that within a cohort of patients’ HNSCC derived primary tumor cell cultures, trametinib showed selective sensitivity in reducing tumor metabolic rate in a subset of HNSCC. Recently, trametinib as a treatment for HNSCC has been supported by clinical data as the result of the Phase II randomized clinical trial of trametinib in HNSCC showing mixed results, with observed reduction in tumor volume in 65% of patients.^11^ These mixed results likely relate to the complexity and heterogeneity of HNSCC. HNSCC is a particularly diverse cancer in terms of molecular classification, biological characteristics, genomics, and HPV status, as well as in anatomical sites.^23^ Our exploration into the landscape of combination therapies offers the opportunity of increasing treatment efficacy amongst this heterogenous population of patients.^24^ Rational optimization of trametinib therapy for HNSCC will be enhanced by further evaluation of the differential targets in HNSCC, pathways underlying sensitivity or resistance, dose selection, and identification of promising synergistic combinations. This approach to new targets and pathway synergies is relevant to other cancers.

In order to further enhance the response of trametinib, we evaluated the synergistic response of both trametinib-sensitive and trametinib-resistant HNSCC patient-derived cell lines. Many cell lines showed drug synergies between VEGFR, PIK3CA, AXL, AKT, and MET inhibition. Using MAPK synergies by co-targeting the AKT, MTOR, and PIK3CA has shown promise in other HNSCC studies.^25,26^ We also observe a statistically significant increase in AKT phosphorylation at 72 hours. This is concordant with dose response immunoblotting on the patient-derived cell lines (Supplementary Fig. S3B). The increase in AKT phosphorylation is a plausible mechanism behind the synergies observed in the PIK3CA and AKT inhibitors in combination with trametinib.

An additional area of interest is the Receptor Tyrosine Kinase (RTK) response in combination with Trametinib, many of which are upstream regulators of the PIK3CA and AKT pathways. AXL and MET have been suggested to increase resistance of MAPKi and have shown synergies with MAPKi in non-HNSCC models.^27^ However, despite the observation of synergy between AXL/MET inhibition and trametinib combination treatment, there seemed to be a reduction in the overall AXL and MET expression in trametinib-treated cells, suggesting that the cells that are sensitive to trametinib may be the same cells that highly express AXL and MET (Supplementary Fig. S8). Alternatively, trametinib may lead to the down regulation of AXL and MET in the context of HNSCC.

Interesting, the cell line demonstrating the most EMT, 10336, was only synergistic with FGFR inhibition.^28^ By identifying the therapeutic window that yields the maximum synergistic benefit and minimum CI value, we identified optimum dosing regimens across many combination therapies (Table S1). Identification of optimal dosing ranges is critical due to off target effects of many of the inhibitors, as well as for limiting drug toxicity.^29^

Additionally, we show that response in vitro correlates to a decrease in tumor volume in vivo. This is especially promising due to the concordance between the primary tumor and the PDX established tumor. Furthermore, our data suggest that treatment durability may be improved by the addition of a synergistic agent, in this case glesatinib. Glesatinib targets MET, TIE-2, AXL, as well as VEGFR 1/2/3. It is unclear which of these signaling pathways may be responsible for a more durable response. Whereas trametinib decreased the concentration of both MET and AXL on the cellular surface in our cell lines shown by immunofluorescence (Supplementary Fig. S8), VEGFR1 showed significant increased affinity at 72 hours in the RPPA data. The implications of this finding remain to be elucidated; however, VEGFR1 as a plausible molecular mechanism behind this synergy and needs to be substantiated in further studies.^30^

These exploratory RPPA studies identified many proteins upregulated in response to trametinib treatment that are targetable, including DDR1, LCK, MMP14, PAI-1, SOD, CD26, and FAK, that could be evaluated for future trametinib combination therapies. It was of interest to observe that trametinib-resistant lines had an increased adaptive proteomic response to trametinib treatment, including the activation of the MAPK pathway though increase of p-MEK1 and GAB2. The totality of these observations is suggestive of an adaptive response to trametinib allowing for resistant cell lines to survive MAPK inhibition. The primary culture conditions selected for epithelial HNSCC tumor cells without fibroblast or immune cells. Both a limitation and an advantage of this approach is that the RPPA data reflect the intrinsic epithelial tumor cells. While this allows us to dissect the intrinsic pathways of the patient’s individual HNSCC, it lacks the context of the tumor microenvironment. Recently, trametinib has been reported to enhance efficacy of PD-L1 inhibitors in HNSCC. Thus, besides combinations for intrinsic targeting, trametinib could be combined with already approved immunotherapies to increase treatment efficacy in HNSCC.^31^

While this study yielded several promising candidate drug combinations for trametinib, a major limitation remains biomarkers predicting who will and will not respond to trametinib or trametinib combination therapies. This is especially important considering that trametinib enhanced metabolism in a subset of primary cultures within our HNSCC patient cohort (Figure 1a). In our investigation of the proteomic correlations of trametinib sensitivity, no single protein predicted significant correlation to drug response. Due to the heterogeneity of the tumors and complexity of the signaling pathways, it is likely that a combination of proteins will be necessary to predict the complexity of drug sensitivities. Culturing HNSCCs to determine responsiveness to trametinib may have a role in determining drug efficacy applicable to the clinic. In this regard, Serial Measurements of Molecular and Architectural (SMMART) trials in several cancers have opened at our institution with patient-derived cells as a component, along with imaging, genomic evaluation, and many other measurement types. The findings reported here offer evidence for trametinib utility in HNSCC and potential synergy with several other drugs.

A major limitation of the study is inability to address the potential complementary role of intrinsic and extrinsic targeting or how intrinsic pathways may affect the microenvironment or immunotherapy responses, given our focus on the epithelial tumor cell component in culture, and our use of an immunocompromised animal model for in vivo assessment of drug response.^32,33^ Further preclinical studies on these combinations in more complex immunocompetent models and on predictive biomarkers in larger cohorts is warranted to extend the therapeutic options of HNSCC patients by applying promising regimens in the clinic.

## Materials and methods

### Cell lines and reagents

Primary tumor cell lines were obtained from patients treated at the OHSU Department of Otolaryngology for HNSCC from 2014 to 2019 (Dermatology Molecular Profiling Resource Repository, IRB #10071). Cell culture methods have been previously described.^24,34^ Primary tumor samples were collected from the operating room in DMEM/F12 Media, supplemented with 2x antibiotic/antimitotic (Invitrogen, Carlsbad, CA). Tissues were washed 3 times in fresh DMEM/F12 Media and minced before plating. Cells were cultured in DMEM/F12 Media (Gibco, 11320082), supplemented with 5% supplemented BCS (Hyclone), 1x antibiotic/antimitotic (Invitrogen, Carlsbad, CA), 1.8 × 10–4 M adenine (Sigma, St. Louis, MO), 0.4 μg/mL hydrocortisone (Sigma), 1 × 10–10 M cholera enterotoxin (Sigma), 2 × 10–9 M triiodothyronine (Sigma), 5 μg/mL insulin (Sigma), and 10 μg/mL epidermal growth factor (Invitrogen). Fibroblasts were removed from primary cultures by differential trypsinization using Trypsin-EDTA (0.25%), phenol red (Gibco, 25200056). Frozen cultured cell lines were used within five passages of the original tumor.

### Trametinib Inhibitor Assay

At time of selection, sensitive cases that were <20% of the median IC50 values were selected for validation. Trametinib (LCLabs, Woburn, Massachusetts, T-8123) was plated using the ECHO liquid handler in a 13, three-fold dilution series ranging from 10 μM to .01 nM. Cell lines 10004, 10292, 10336, 10326, and 20003 were randomized by column and plate order and plated in triplicate. Cells were assessed for metabolism after 72 hours of incubation at 37°C using atetrazolium-based metabolic rate assay (MTS, Promega, Madison, WI, PR-G3581) by measuring absorption at 490 nM using a Biotek Synergy H1 Microplate Reader. Values were then normalized to wells that received no drug. Dose response curves were created for area under the curve (AUC) analysis.

### Combination Assays

Drug combinations were chosen based on preliminary combination data, drugs with specific targeted pathways, and targets from siRNA data. Trametinib-combination therapies were chosen for further validation on custom 7 × 7 drug combination matrices created using ECHO liquid handler. Trametinib started at 0.03 μM (while all other combination drugs started at 1 μM) and followed 6 three-fold dilutions. Cells were assessed for metabolism after 72 hours of incubation at 37°C using atetrazolium-based metabolic rate assay (MTS, Promega, Madison, WI, PR-G3581) by measuring absorption at 490 nM using a Biotek Synergy H1 Microplate Reader. Values were then normalized to wells that received no drug.

### Drug Combination Analyses

In parallel to the Bliss beta coefficients calculated by the SynScreen program, we evaluated synergy using the Chou-Talalay Combination Index (CI).^35,36^

$$CI=\frac{C_{xx}^{A}}{IC_{xx}^{A}}+\frac{C_{xx}^{B}}{IC_{xx}^{B}}$$

Where $C_{xx}^{A}$,$C_{xx}^{A}$ are the concentrations of drugs A, B, respectively, in combination when inhibition is xx%.

Single-agent dose–response curves were fitted with the Hill equation^37^ using a gradient-descent optimizer. Combination dose–response observations were initially modeled as a multidimensional logistic regression; however, after manual inspection, we noticed that the combination-agent dose–response surface exhibited complex behavior that the logistic model was not capable of explaining. To address this, we chose a non-parametric Gaussian process (GP) model.^38,39^ Manual inspection of the GP explained more variance in the dose-response without overfitting within the measured dose-range. The GP parameters were chosen using evidence lower bound (ELBO) and a gradient-descent optimizer.

Several single-agent inhibitors did not create the desired effect (50% inhibition) within the measured concentration range. To address this, single agents that did not reach 50% inhibition were assigned the inequality:

$$IC_{50}\geq \max-conc$$

Where max−conc is the maximum concentration of the measured dose range. This IC50 inequality then propagates through to the CI calculations as:

max−conc

If the desired effect (50% inhibition) was not reached in combination screen, then no CI values were calculated.

### Immunoblotting

Patient-derived tumor cells were treated with cell lysis buffer (Cell Signaling Technologies, Danvers, Massachusetts, 9803S), complete mini protease inhibitor mixture tablets (Roche, 11836153001), and PhosSTOP Phosphatase Inhibitor Cocktail Tablets (Roche, 4906845001). Lysates were spun at 8,000 xg for 10 minutes at 4°C, mixed 3:1 with 4x Laemmli Sample Buffer (Bio-Rad, 1610747) with b-ME, and heated at 95°C for 5 minutes. Lysates were run on Criterion TGX Precast Midi Protein Gel (Bio-Rad, 5671083), transferred to a polyvinylidene difluoride membrane (Bio-Rad, 1704157), and blocked for 1 hour in TBS-T with 5% milk. Blots were probed overnight at 4°C with PERK (C33E10) Rabbit, Phospho-p44/42 MAPK (Erk1/2) Rabbit, Phospho-Akt (Ser473) (D9E) XP Rabbit, Akt (pan) (C67E7) Rabbit, or a-Tubulin (DM1A) Mouse (Cell Signaling Technology). HRP conjugated secondary antibodies, anti-rabbit, or anti-mouse IgG were used for rabbit and mouse primary antibodies, respectively. Blots were developed using Clarity^TM^ or Clarity Max^TM^ Western ECL Substrate (Bio-Rad, 1705060 and 1705062) and imaged using a Bio-Rad ChemiDoc touch MP Imaging System. For all western blots the same blot was probed repeatedly.

### PDX Experiments

20 female NOD SCID Gamma (NSG) mice (8–16 weeks old) were used for this study (Jackson Laboratory, Bar Harbor, Maine). Mice were inoculated with 2 million 10004 HNSCC cells in right flank, and measurements of tumor growth were taken daily. Tumors were measured, and volume was calculated by Length*Width^2^/2.^39,40^ Mice were randomized using R, into vehicle, trametinib, and trametinib + glesatinib treatment groups. Treatment of 1 mg/kg/day trametinib (LCLabs, T-8123) (n = 6) or 60 mg/kg/day glesatinib (Chemitek, CT-MG265) and 1 mg/kg/day of trametinib (n = 6) or vehicle (DMSO) control (n = 8) was delivered via oral gavage once tumors reached 350 mm^3^. Glesatinb treatment was lowered from 60 mg/kg/day (weeks 8–10) to 40 mg/kg/day (week 10), to 15 mg/kg/every-two-days (week 11), and finally down to 9 mg/kg/day every-two-days (week 13) after combination toxicity was observed. Three mice died during dose de-escalation in the combination group. All mouse cohort number changes are displayed in Figure 3. Trametinib treatment was halted during week 13 and was then continued during week 14 for the course of the treatment. Data analyses were stopped after the first tumors were harvested at tumor volume of ~2000 mm^3^. Animals were housed within the same treatment group to avoid treatment contamination. Groups were non-blinded throughout the experiment and analyses. Graph pad prism was used to perform student’s t-tests and to generate figures. All studies were performed according to guidelines approved by OHSU Institutional Animal Care and Use Committee.

### Immunofluorescence

Cells plated in 12-well vessels on collagen coated coverslips, fixed with 4% formalin solution at room temp for 15 minutes, washed twice with PBS, permeabilized in PBS + 0.1% TritonX. Cells were incubated overnight at 4°C with primary unconjugated rabbit cytokeratin 5 (abcam, Cambridge, United Kingdom, ab52635), mouse vimentin (abcam-ab8978), and Guinea pig cytokeratin 8/18 (Fitzgerald, Acton, Massachusetts, 20 R-Cp004) in blocking buffer composed of 2% BSA, 5% goat serum, and PBS. Cells were washed three times in PBS-T and incubated with mouse, rabbit, and goat secondary antibodies labeled with 488 GFP, 594 Texas, 647 Cy5, respectively, and diluted 1:300, along with a DAPI. Cells were washed twice with PBS-T and imaged with the 10x objective on the EVOS microscope. Image segmentation, storage, and data analyses were performed using Image J software.

### Reverse phase protein array (RPPA)

Cell lysate was collected using RPPA lysis buffer provided by MD Anderson Cancer Center RPPA Core and following their collection protocols.^41^ Cells were grown in 6-well dishes, and cell lysate was collected when cells reached confluence of 50–90%. Lysate buffer and SDS were purchased from MD Anderson. Cell lysate was immediately diluted to ~1 μg/μl and stored at −20°C prior to shipment to MD Anderson. Immune blots of excess lysate confirmed molecular response. Cells plated in triplicate in 96-well dish confirmed functional response after incubation for 72 hours using Promega MTS assay.

### RPPA Analyses

The MD Anderson RPPA Core Facility performed the RPPA and processed all data. Full annotation of the antibodies can be found at the MD Anderson RPP Core Facility website: https://www.mdanderson.org/research/research-resources/core-facilities/functional-proteomics-rppa-core/antibody-information-and-protocols.html. For all analyses, we considered the “validated” antibodies only, as defined by MD Anderson and used the normalized log2 values.^41^ We performed a differential affinity analysis comparing trametinib versus DMSO for both the 24-hour and 72-hour treatment groups. We used the Bioconductor package limma and defined an antibody to be significant if the FDR < .05 using the Benjamani-Hochberg correction.^42^ In addition, we performed differential affinity analysis comparing samples treated with DMSO and trametinib separately for resistant and sensitive patients. We used the Bioconductor package limma and defined an antibody to be significant if the FDR < .05 using the Benjamani-Hochberg correction.^42^

## Ethics Statement

Oral and written patient consent was obtained under OHSU IRB protocol 10071, and work was performed under OHSU IRB protocol 809. All animal experimentation was approved by the Institutional Animal Care and Use Committee (IACUC) of OHSU protocol 1489.

## Supplementary Material

Supplemental Material

## Data Availability

Further documentation of these methods, data and analysis can be found here: https://github.com/nathanieljevans/HNSCC_Trametinib_Combination_Therapy
