# Supplementary material for: Functional proteomics of patient derived head and neck squamous cell carcinoma cells reveal novel applications of trametinib
Source: Cancer Biol Ther. 2022 Mar 28;23(1):310–8. doi: 10.1080/15384047.2022.2055420 (PMC8966983; doi:10.1080/15384047.2022.2055420)
Supplement: Supplemental Material [file KCBT_A_2055420_SM5018.docx]

**Supplementary Figures**

**Figure S1** (A) Area under the curve values for the average HNSCC cell line, 10308 tumor cell line, and 10309 matched normal cell lines response to trametinib. The red line demarcates no effect. (B) HNSCC Cohort AUC values in response to trametinib. Red line demarcates median cell line response. (C) 10004 cells were treated for 72 hours with trametinib or TAK-773.


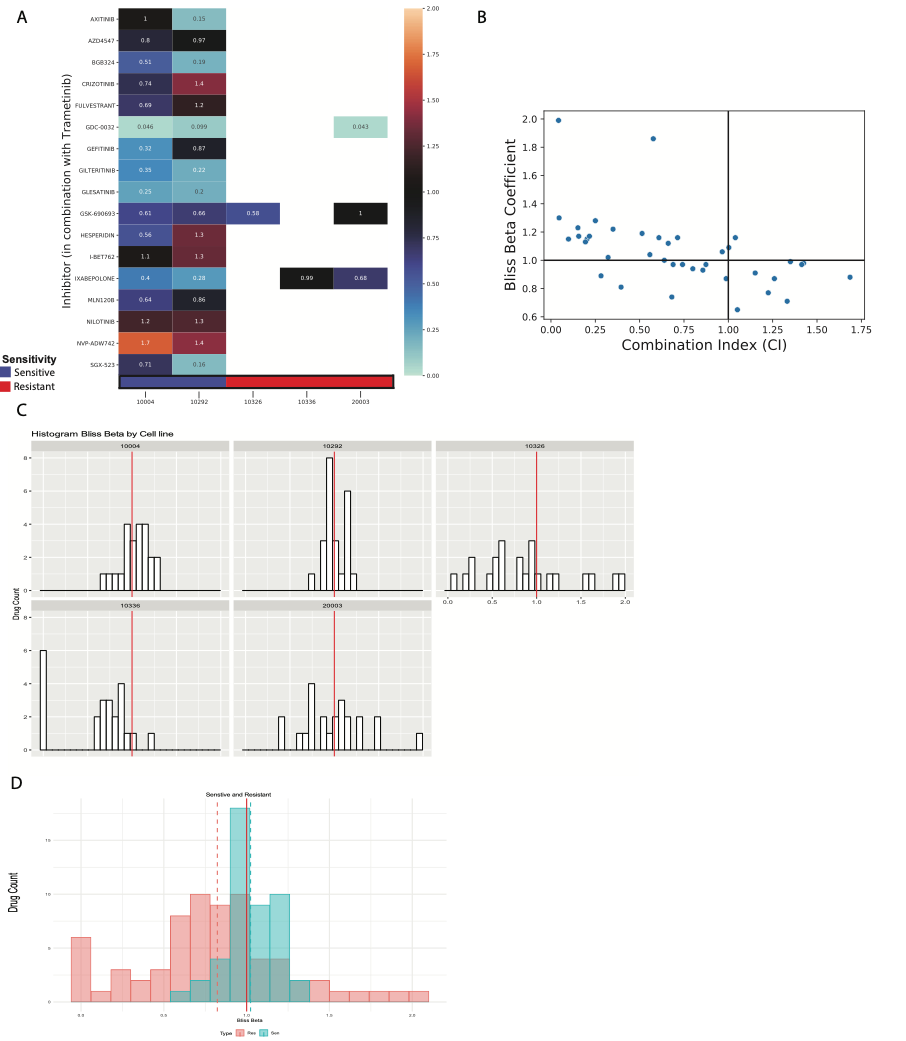


**Figure S2** (A) Minimum CI values for each cell line treated in combination with trametinib. Values < 1 indicate synergistic relationship, whereas values > 1 indicate antagonistic relationship. Values are calculated using IC50 values; for agents that did not reach 50% inhibition, no CI could be generated. (B) Spearman correlation between Bliss beta coefficients and CI values were -.65 (C) Histogram of Bliss beta values stratified by cell line with the vertical red line indicating the mean for each cell line. (D) Histogram of Bliss beta values stratified by sensitive (blue) and resistant (red), with the vertical red line indicating the mean for all cell lines, while the dashed line representing the means of the groups.

**Figure S3** (A) The effect of trametinib on phosphorylation of ERK normalized to alpha tubulin expression. Blue coloration indicated trametinib functional sensitivity, while red indicates trametinib functional resistance. (B) Corresponding western blots of 2 sensitive and 2 resistant cell lines. The data point at .001 μM in the 10004 cell line was censored due to observed paradoxical activation of MAPK pathway.

**Figure S4** Patient derived HNSCC tumor cells were exposed to glesatinib in a 6, 3-fold dilution series at a max concentration of 1 μM.

A.


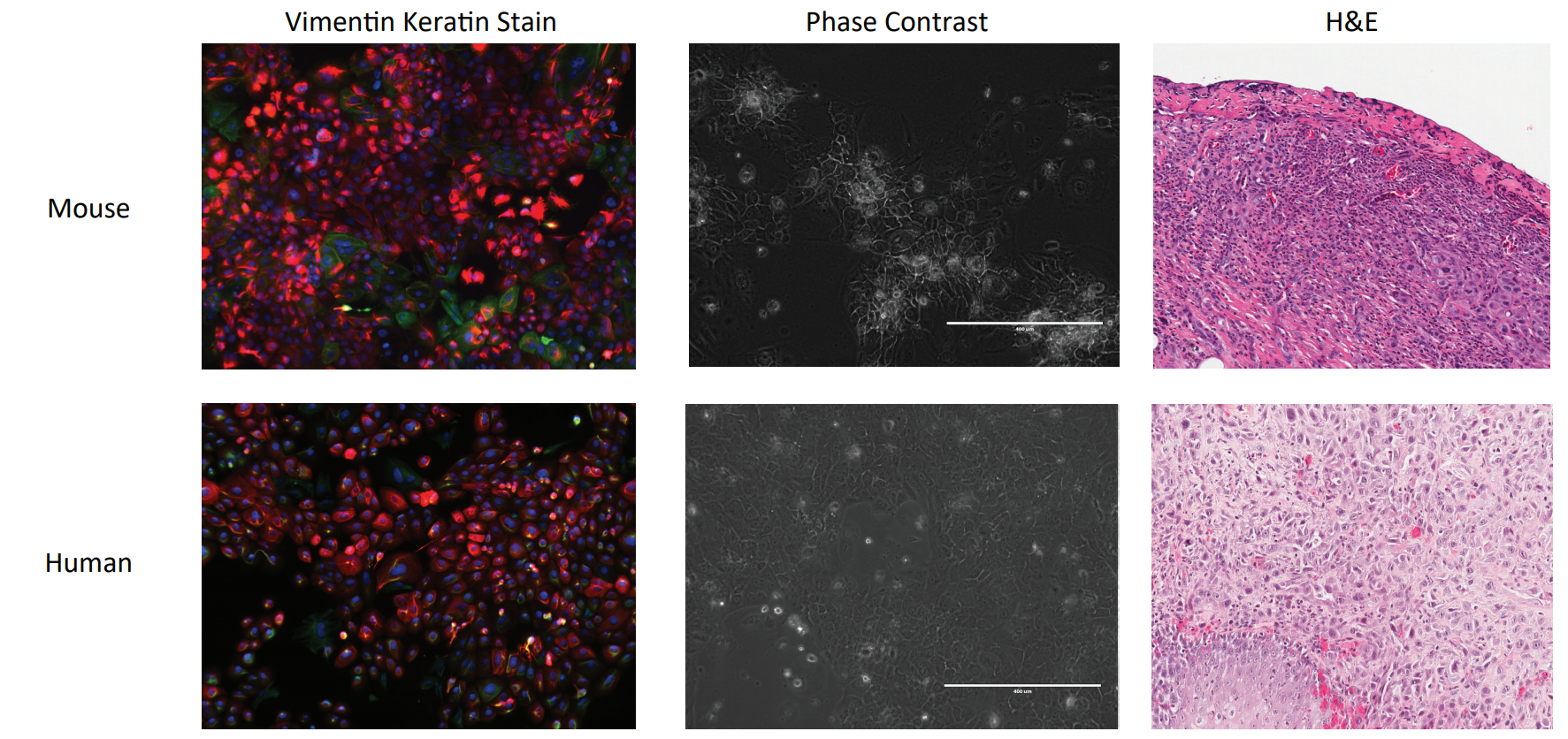


B.
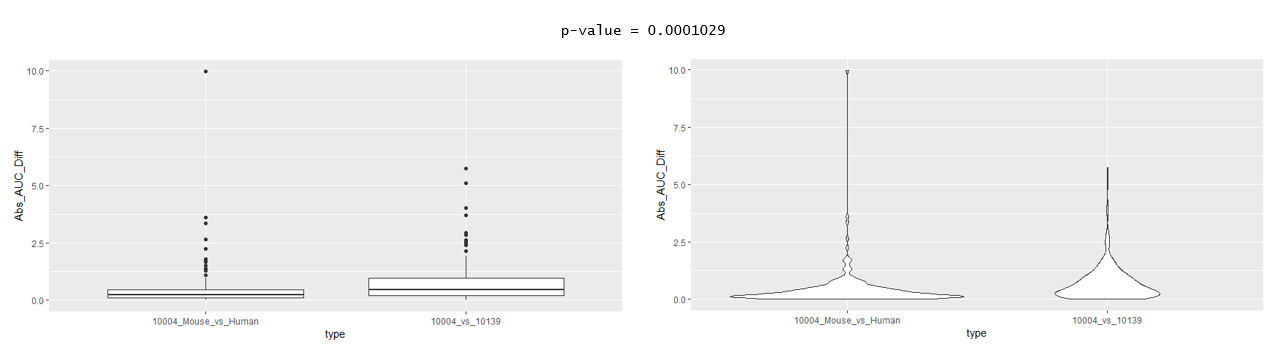
**Figure S5** (A).10004 primary human and mouse explants grown in culture show similar keratin positivity, morphology, and histology. Both mouse and human cells were found to be over 94% keratin positive, representing a less than 6% fibroblast presence in either culture. Cells demonstrated similar epithelial to mesenchymal characteristic, 4% in the original primary tumor cells and 3.1% in the mouse xenograft derived cells, showing dual positivity for vimentin and keratin. Tumors for mouse and human show similar epithelial morphologies. Both H&E of primary tumor and mouse tumors show presence of HNSCC in situ. (B) Mouse cells grown from tumor explants show similar drug sensitives when compared to two different human derived cell lines. Two different primary tumor cell lines show dissimilar drug sensitivities (P value < .0002). Cells coming out of the xenograft are not statistically different in AUC value from cells re-plated on inhibitor assay (P value = .1).

**Figure S6** Cell lines were treated in triplicate with trametinib at IC50 values show functional decreases in the sensitive lines but not in the resistant lines (left). Molecular hitting of the target was confirmed for all lines except in the 10292 due to low treatment concentrations (middle). Cell morphologies were taken for each line for each treatment group (right).

**Figure S7** RPPA normalized relative protein expression for DMSO- and trametinib-treated cell lines for “Caution” antibody MAPK_PT202_Y204 at both 24 and 72 hours.


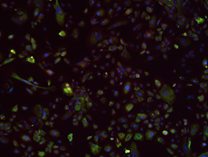

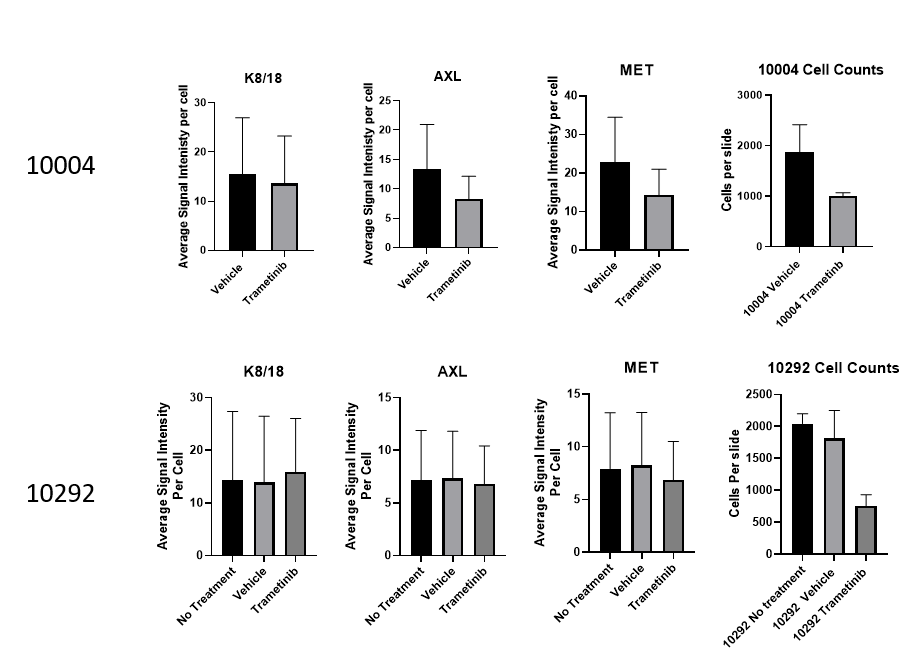


**Figure S8** Representative immune fluorescence of cells treated with trametinib (left). (Green) Cytokeratin, (Texas Red) MET, (Cy5) AXL expression. Trametinib shows an expected decreased number of cells as represented by the DAPI stain. Representative bar graphs of immune florescent quantification (right).
